# Fluorescence of Dyes in Solutions with High Absorbance. Inner Filter Effect Correction

**DOI:** 10.1371/journal.pone.0103878

**Published:** 2014-07-29

**Authors:** Alexander V. Fonin, Anna I. Sulatskaya, Irina M. Kuznetsova, Konstantin K. Turoverov

**Affiliations:** 1 Laboratory for Structural dynamics, Stability and Protein folding, Institute of Cytology Russian Academy of Science, St. Petersburg, Russia; 2 Department of Biophysics, St. Petersburg State Polytechnical University, St. Petersburg, Russia; Russian Academy of Sciences, Institute for Biological Instrumentation, Russian Federation

## Abstract

Fluorescence is a proven tool in all fields of knowledge, including biology and medicine. A significant obstacle in its use is the nonlinearity of the dependence of the fluorescence intensity on fluorophore concentration that is caused by the so-called primary inner filter effect. The existing methods for correcting the fluorescence intensity are hard to implement in practice; thus, it is generally considered best to use dilute solutions. We showed that correction must be performed always. Furthermore, high-concentration solutions (high absorbance) are inherent condition in studying of the photophysical properties of fluorescent dyes and the functionally significant interactions of biological macromolecules. We proposed an easy to use method to correct the experimentally recorded total fluorescence intensity and showed that informative component of fluorescence intensity numerically equals to the product of the absorbance and the fluorescence quantum yield of the object. It is shown that if dye molecules do not interact with each other and there is no reabsorption (as for NATA) and spectrofluorimeter provides the proportionality of the detected fluorescence intensity to the part of the absorbed light (that is possible for spectrofluorimeter with horizontal slits) then the dependence of experimentally detected total fluorescence intensity of the dye on its absorbance coincides with the calculated dependence and the correction factor for eliminating the primary inner filter effect can be calculated on the basis of solution absorbance. It was experimentally shown for NATA fluorescence in the wide range of absorbance (at least up to 60). For ATTO-425, which fluorescence and absorption spectra overlap, the elimination of the primary and secondary filter effects and additional spectral analysis allow to conclude that the most probable reason of the deviation of experimentally detected fluorescence intensity dependence on solution absorbance from the calculated dependence is the dye molecules self-quenching, which accompanies resonance radiationless excitation energy transfer.

## Introduction

Fluorescence is one of the most common and popular methods for studying various biological objects. The method is used to detect, determine the concentration and examine the structure, stability, folding, function and interactions of biological objects. Fluorescence methods are used in all areas of life sciences to study objects ranging from molecules and their complexes to cells and tissues. These studies can use a great variety of fluorophores, including the intrinsic groups of the studied biological objects (e.g., tryptophan residues of proteins or the fluorescent protein fluorophore Cro), the fluorescent probes that specifically bind to the studied biological objects (e.g., ANS or thioflavin T (ThT), its analogs and derivatives) or fluorescent dyes that can be chemically linked to the studied biological objects (e.g., thiol-reactive dyes or amine-reactive dyes).

A significant obstacle to the use of fluorescence methods is the nonlinear dependence of the fluorescence intensity on the concentration of the fluorescent substance. This effect, known in the literature as the inner filter effect, greatly complicates the record of fluorescence excitation spectra and determination of the binding parameters of fluorescent dyes to receptors and constants of fluorescence quenching by external quenchers; this effect often leads to incorrect uses of the method even by experienced researchers. In the literature, the inner filter effect is divided into the “primary inner-filter effect”, which is caused by the absorption of exciting light such that a less intense light flux reaches each subsequent layer of the solution than the previous one, and the “secondary inner-filter effect”, which is caused by the reabsorption of fluorescence [1].

Many attempts have been made to compensate for the inner filter and to linearize the dependence of the fluorescence intensity on the concentration of the fluorescent substance [1]–[18]. The first relation for a correction factor was proposed by Parker and Barnes [2], although it was not clearly derived in their paper. Later, practically the same relation was derived more strictly by Holland et al. [6]. While differing in some details, the proposed relations have a common disadvantage because they include parameters that cannot be accurately determined, namely, the excitation window parameters, which are determined by the masking apertures at the emission cell wall or some other limiting aperture in the emission beam. The proposed relations were shown to be successful up to *A* = 2.0. Yappert and Ingle analyzed these relations in detail [4]. Although the simplification of the correction factor facilitates its use, the simplification limits its scope [1], [19]. There is a settled opinion that fluorescence measurements must be performed in solutions using low concentrations of fluorescent substrates, where the intensity of the fluorescence is proportional to the concentration of the fluorescent substance and the inner filter effect can be neglected. In this paper, we show that this suggestion does not solve the problem. Moreover, to study the interactions of biologically important macromolecules in conditions similar to *in vivo* conditions, it is in principle necessary to work with high concentrations of fluorescent substances, including the presence of other highly absorbing compounds.

In this paper, we offer a new correction method for experimentally recorded fluorescence intensity. As primary and secondary inner filter effects have different physical basis we considered them one by one. We started with the primary inner filter effect because it is always present, even in dilute solutions. For examination the inner filter effect we chose NATA as a target object because this dye has large Stokes shift and, consequently, fluorescence reabsorption (secondary inner filter effect) is negligibly small. We showed that total fluorescence can be presented as a product of correction factor and the informative component, which numerically equals to the product of the absorbance and the fluorescence quantum yield of the object. The correction factor depends only on solution absorbance and can be determined experimentally for any spectrofluorimeter in its work range of absorbance. It was shown that for spectrofluorimeter with horizontal slits the calculated correction factor can be used in a wide range of absorbance. It appeared that slits orientation is crucial factor in the device capacity to detect fluorescence of solution with high absorbance. Fluorescence of solutions with high (up to 150) absorbance was experimentally detected using Cary Eclipse spectrofluorimeter. After elaboration the approach for correcting primary inner filter we turned to the study fluorescent dye ATTO-425, which absorption and fluorescence spectra overlap. We corrected its fluorescence intensity firstly on the primary inner filter effect and then on the secondary inner filter effect. It was shown that after this correction the dependence of total fluorescence intensity though approached to the calculated plot did not coincide with it. Analysis of ATTO-425 spectral characteristics showed the molecules self-quenching, which accompanies resonance radiationless excitation energy transfer.

## Materials and Methods

The fluorescent dye ATTO-425 from ATTO-TEC (Germany), N-acetyl tryptophan amide (NATA) from Sigma (USA) and PBS buffer from Sigma (USA) were used without further purification. ATTO-425 was dissolved in PBS buffer (pH 7.4), and NATA was dissolved in distilled water.

Fluorescence measurements were performed in a homemade spectrofluorimeter [20] and Fluorolog-3 (Horiba, Japan), which have vertical slits and a Cary Eclipse spectrofluorimeter (Agilent Technologies, Australia), which has horizontal slits. Fluorescence measurements in the Fluorolog-3 (Horiba, Japan) were performed in the Resource Center for Optical and Laser Materials Research of St. Petersburg State University, Russia. The fluorescence emission and excitation spectra of NATA were recorded at *λ_e_*_x_ = 280 nm and *λ_em_* = 350 nm, respectively. The fluorescence excitation spectra of ATTO-425 were recorded at different wavelengths of emission in the range from 470 nm to 550 nm with a step of 10 nm. The fluorescence spectra of ATTO-425 were recorded with *λ_ex_* = 436 nm, corresponding to the maximum of the long-wavelength band of the dye.

All the concentrational dependences for NATA and ATTO-425 were measured at constant experimental conditions (the excitation slit and emission slit values, the scan speed and the setting of PMT Detector Voltage). In particular for Cary Eclipse spectrofluorimeter excitation and emission slits were 5 nm, the scan speed was 600 nm/min, PMT Detector Voltage was in the range 410–530 V. Once chosen it was unchanged for measurement all spectra for plotting the dependence of *F*(*λ_ex_*) on *A_FL_*. All experiments were performed in a cell of dimensions 10×10×4 mm Starna Cells, Inc (USA).

The fluorescence quantum yield of ATTO-425 was taken as 0.9 (Product cataloque 2013/2015, ATTO-TEC GmbH, Germany), and the fluorescence quantum yield of NATA was taken as 0.14 [21]. The absorption spectra were recorded using a U-3900H spectrophotometer (Hitachi, Japan) in cells from Hellma GMBH & Co. (Germany) with different optical path lengths: 10, 1, 0.1 and 0.01 mm (100-QS 10, 100-QS 1, 106-QS 0.1 and 106-QS 0.01 with cell holder 013.000).

## Results and Discussion

### Physical essence of the fluorescence intensity corrected for the inner filter effect: the product of the absorbance and the fluorescence quantum yield

The nonlinearity of the dependence of the fluorescence intensity on the concentration of a fluorescent substance is caused by the so-called primary inner filter effect. The reasons for this effect are the attenuation of the exciting light flux on its path through an absorbing solution (Beer–Lambert law) and the difference between the area that is illuminated by the exciting light and the working area from which the fluorescence light is gathered. In the ideal case when these areas coincide, the recorded total fluorescence intensity 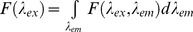 is proportional to the fraction of exciting light absorbed by the solution (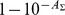):
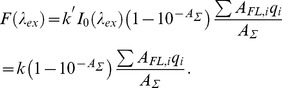
(1)Here, 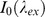 is the intensity of the excitation light at *λ_ex_*, *k′* is a proportionality factor, 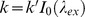, *A_Σ_* is the total absorbance of the exciting light in the solution, 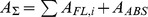, 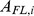 and 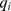 are the absorbance and fluorescence quantum yield of the *i-*th fluorescent component, respectively, *A_ABS_* is the total absorbance of the nonfluorescent components. 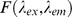 is fluorescence intensity excited at *λ_ex_* and recorded at *λ_em_*, so that fluorescence spectrum is 
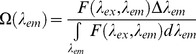
.

**Figure 1:**
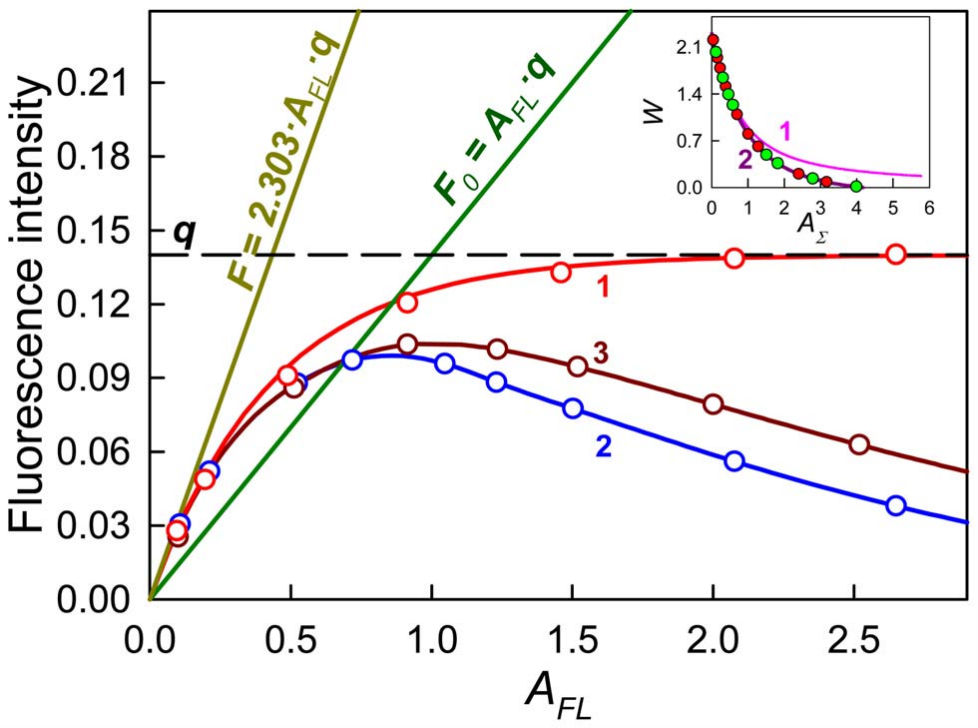
The dependences of the total fluorescence intensity of N-acetyl tryptophan amide (NATA) on its absorbance. Curve 1 (solid line) represents the fluorescence intensity that was calculated according to Eq. 2; the circles on the curve represent the fluorescence intensity values recorded by the Cary Eclipse (Agilent Technologies, Australia) spectrofluorimeter. Curves 2 and 3 represent the fluorescence intensity recorded by a homemade spectrofluorimeter [20] and by a Fluorolog-3 (Horiba, Japan) spectrofluorimeter, respectively. The straight line *F* = 2.303*A_FL_q* is tangent to curves 1, 2 and 3 at *A_FL_* = 0. Here *k′* is chosen so that *k′I_0_* = 1, and consequently 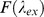 numerically equals to *q* at 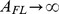. The **inset** represents the dependence of *W* on *A_FL_* calculated by Eq. 4 (curve 1) and determined experimentally for the homemade spectrofluorimeter (curve 2). For this plot, dyes with different fluorescence quantum yields were used: quinine sulfate (*q* = 0.52 [36], green circles) and NATA (*q* = 0.14 [21] red circles).

In the simplest case (*A_ABS_* = 0, *i* = 1), *A_Σ_* = *A_FL_*, and consequently, Eq. 1 will become the following [2]:
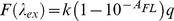
(2)It is easy to show that fluorescence intensity of a solution can be presented as a linear function of *A_FL_* with a slope of *q* (Figure 1):
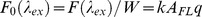
(3)Here, *W* is a correction factor:
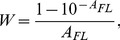
(4)which tends to 2.303 as 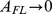.

It should not be surprising that 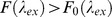 for 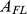 which satisfies the condition 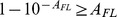 (Figure 1). This is the result of the selection of coefficient *k* so that the value of the corrected fluorescence intensity corresponds to the simple physical essence of the product of the absorbance and the fluorescence quantum yield. The slope of the tangent of the dependence 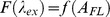 at 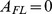 is 2.303 times greater than the slope of the dependence 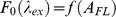 (Figure 1).

### Fluorescence intensity correction on the primary inner filter effect

In the majority of spectrofluorimeters (except as we know for the Cary Eclipse spectrofluorimeter, see below), the area illuminated by the exciting light does not coincide with the working area from which the fluorescence light is gathered. As a result, the detected fluorescence intensity is not proportional to the portion of light absorbed by the solution, and the correction factor cannot be calculated according to Eq. 4.

Starting with Parker and Barnes [2], the authors of subsequent works on the inner filter effect [4], [6], [8] tried to linearize the dependence of the fluorescence intensity on the absorbance by introducing correction factors that depends on the difference between of the area of solution that is illuminated by exciting flux and the area from which the fluorescence is gathered. However, this task is very difficult, if at all feasible. In some works that refer to Lakowicz [19], it is considered that the fluorescence light is gathered from the center of the cell. This assumption certainly simplifies the expression, although it is far from reality. The authors of many experimental works (see, e.g., [22]–[24]) either do not account for the inner filter effect or, in accordance with Parker and Rees [25], believe that the fluorescence intensity in very dilute solutions is proportional to the concentration of the fluorescent substance. In actuality, according to Eq. 2, 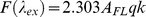 at 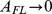 (Figure 1). However, it should be kept in mind that this relationship is equation of the tangent at *A_FL_* = 0 to the recorded (and calculated according to Eq.2) dependence and that these curves coincide only in one point: *A_FL_* = 0. Even at *A_FL_* = 0.1, the difference between recorded (calculated according to Eq. 2) dependence and its tangent (at *A_FL_* = 0) is 12%, and at *A_FL_* = 0.3, the difference is 38%! Moreover, the use of diluted solutions hardly seems acceptable if a high-absorbance solution is an inevitable experimental condition, e.g. when the interactions of fluorescent molecules at high concentrations or in the presence of other absorbing molecules are studied. To perform such examinations, the fluorescence intensity in the broadest possible range of concentrations (absorbance) must be able to be recorded while other conditions of the experiment remain constant.

#### Experimental determination of the dependence of W on AFL for used spectrofluorimeter

In the present work we propose a new way of linearization of the experimentally recorded dependence of fluorescence intensity on absorbance. For anyone spectrofluorimeter the dependence of *W* on *A_FL_* may be determined experimentally with the use of a standard, which molecules do not interact with each other and for which quantum yield (*q_ST_*) is known [26]–[28].

**Figure 2:**
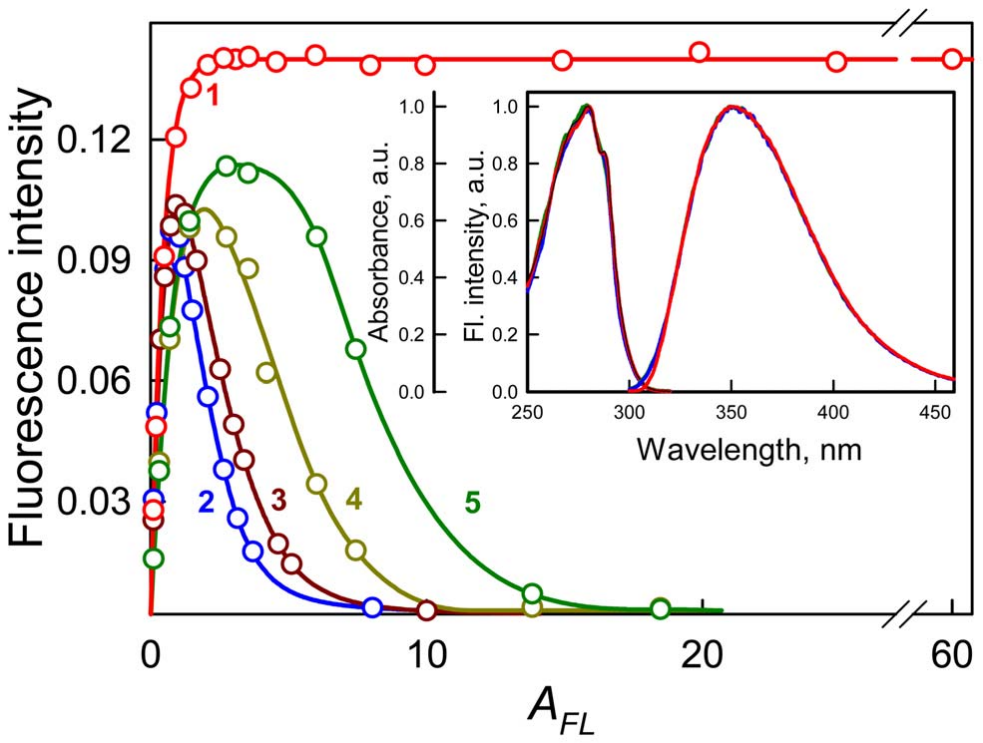
The dependence of the total fluorescence intensity of N-acetyl tryptophan amide (NATA) on its absorbance at high concentrations of fluorophore, as recorded by a Cary Eclipse spectrofluorimeter. Curves 1, 2 and 3 are the same as in Figure 1. Curves 4 and 5 represent the fluorescence intensity recorded with the use of microcells (5×5 mm cross section) and in triangular cell. The absorbance was determined using a Hitachi U-3900H spectrophotometer in cells with different optical path lengths. **Inset:** The corrected and normalized fluorescence excitation and emission spectra of NATA. The fluorescence excitation and emission spectra were recorded by a Cary Eclipse spectrofluorimeter at *λ_em_* = 350 nm and *λ_ex_* = 280 nm, respectively. The correction was performed according to Eq. 4. The red, blue and green curves correspond to *A_280_* = 60, 1.5 and 0.2, respectively. The black curve is the absorption spectrum.

According to Eq. 3 *W_exp_ (A_FL_)* can be determined as follows:
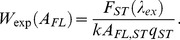
(5)Here 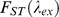 and *A_FL,ST_* are recorded fluorescence and absorbance of standard solution. We can determine analytical form of the function as:
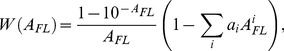
(6)where the first part of the product is correction factor, which is used if total fluorescence intensity is proportional to the fraction of light absorbed by solution, and the second part of the product is additional correction factor, which account non-coincidence of the region illuminated be excitation flux and working region from which fluorescence light is gathered. The values of *k* and *a_i_* can be found by minimization of functional:
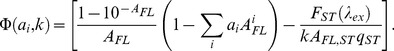
(7)The number of polynomial terms *i* is determined by the statistically reliable fit of the search function to the experimental data. The dependence of factor *W* on *A_FL_* determined for anyone spectrofluorimeter allows to correct the recorded fluorescence intensity. This dependence is characteristic of the used spectrofluorimeter. It is not needed to determine it over again in each new experiment. Such correction is effective in all range of 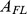 where fluorescence can be reliably measured. However, for the majority of spectrofluorimeters this region is rather narrow. In standard cells with a 10×10 mm cross section, the fluorescence cannot be measured for 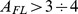 in principal (Figures 2, curves 2 and 3). The use of microcells with a 5×5 mm cross section instead of standard cells or the measurement of the fluorescence from the front wall of the cell (measurement in triangular cells) reduces the inner filter effect, thereby increasing the range of concentrations of the solutions which fluorescence can be recorded (Figure 2, curves 4, 5). Though these adaptations enlarge the working range of absorbance where fluorescence can be detected, it is significantly smaller than that provide Cary Eclipse spectrofluorimeter (see below). Furthermore, the use of triangular cells complicates the elimination of the excitation light reflection and scattering that makes practically impossible detection of fluorescence intensity of a dye at low concentrations. The use of microcells significantly diminishes sensitivity of the device.

### Peculiarities of the Cary Eclipse spectrofluorimeter: the possibility of work with solutions of very high absorbance

We showed experimentally that unlike all known spectrofluorimeter, Cary Eclipse spectrofluorimeter allows to record fluorescence of solutions with very high absorbance. It was shown that using this spectrofluorimeter one can reliably detect the fluorescence signal from NATA solutions with absorbance at *λ_ex_* even greater than 150 (data not shown). Fluorescence intensity of NATA solution with *A* = 136 deviated from calculated value for 10%. Probably, it is due to the interactions of NATA molecules with each other, as such concentrations are near the limit of NATA solubility. For NATA the experimentally recorded dependence of the fluorescence intensity on the absorbance of the fluorescent substance coincides with the dependence calculated according to Eq. 2 with deviation less than 1% at least up to *A_FL_* = 60 (Figures 1 and 2, curve 1). The coincidence of experimentally recorded dependence of total fluorescence intensity with that calculated according to Eq. 2 is possible only in the range of absorbance where two conditions are simultaneously satisfied:

- the used spectrofluorimeter provides the proportionality of the detected fluorescence intensity to the part of the light absorbed by the fluorescent solution;
- the NATA molecules do not interact with each other neither by direct coupling (dimers or excimers formation), nor by light reabsorption (secondary inner filter effect), nor by resonance nonradiative energy transfer, which leads to self-quenching, as overlap of their absorption and fluorescence spectra is negligibly small.

Each of these conditions is a necessary but not sufficient for the coincidence of experimentally recorded dependence of total fluorescence intensity with that calculated according to Eq. 2. The choice of NATA as a reference compound was successful primarily due to its large Stokes shift of fluorescence spectrum and, consequently, there were no effects associated with reabsorption of fluorescence and non-radiative resonance energy transfer from the excited molecule to molecule in the ground state (see below for the results of fluorescent dye ATTO-425 study). Our results allow to use Cary Eclipse spectrofluorimeter to study the interaction of the dyes molecules in a large range of concentration (up to *A_FL_* = 60), and to use for fluorescence correction the value of *W* calculated according to Eq. 4.

#### What is the fundamental difference between Cary Eclipse spectrofluorimeter from all others?

**Figure 3:**
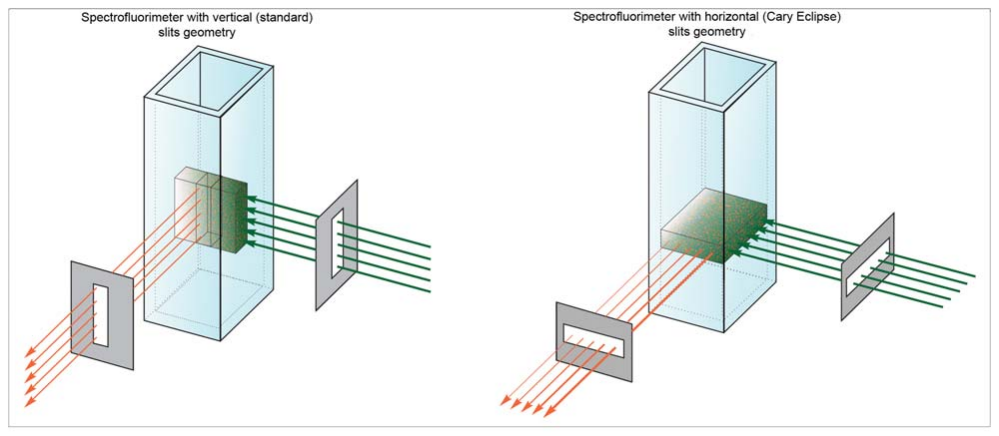
Schematic presentation of the light fluxes in the spectrofluorimeters with vertical (standard) and horizontal slit geometries. The figure was constructed on the basis of the advertising material by Steensrud [30]. In both cases, the slits are projected to the center of the cell.

We suggested that the principal difference of Cary Eclipse spectrofluorimeter in comparison with others is its slits configuration: Cary Eclipse spectrofluorimeter has horizontal slits while all others have vertically slits (Figure 3).

Surprisingly, there is very few information on this feature of the Cary Eclipse spectrofluorimeter. The only mention we found of this design feature of the device was in the work by Jameson et al. [29]. Recently, some advantages of such a slit geometry were described in the advertising material “Selecting the correct spectrophotometer for quality results” by Steensrud [30]. However, this presentation reports high sensitivity and possibility to work with highly diluted solutions, but does not highlights its benefit of using solutions with high absorbance.

Our data show that even for *A_FL_* = 60, when 99.9% of light is absorbed by 0.5 mm of solution, the recorded fluorescence intensity is proportional to *A_FL_ q*. It means that the light fluxes configuration provide the coincidence of the area illuminated by the excitation light with the area, from which the fluorescence light is collected. Thus at least up to *A_FL_* = 60, the efficiency of fluorescence light collection does not depend on how far in the solution penetrates the exciting light. Any deviation of the experimentally recorded dependence of tested dye total fluorescence on its absorbance using spectroflurimeter Cary Eclipse from the dependence calculated using Eq. 2, will suggest some interactions of the dye molecules. Extra studies allow determining the type of interaction of dye molecules in the solution with its high concentration.

### The study of dye molecules interaction in solutions of high absorbance. Fluorescent dye ATTO-425

#### Fluorescence correction on the primary inner filter effect

**Figure 4:**
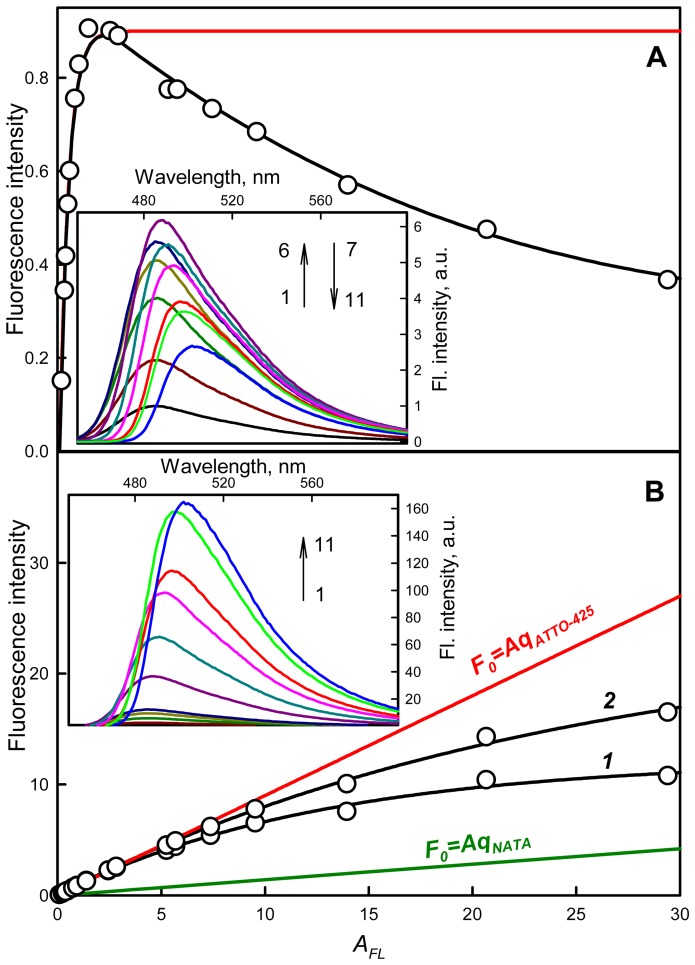
The dependence of the total fluorescence intensity of ATTO-425 on the solution absorbance. On Panels A and B the red curves *F(A_FL_)* and *F_0_(A_FL_)* represent the dependences of the total fluorescence intensity on the solution absorbance calculated according to Eq. 2 and Eq. 3 (*q* = 0.9), respectively. The dependence of the ATTO-425 fluorescence on its absorbance recorded by a Cary Eclipse spectrofluorimeter is given on Panel A (black curve); this dependence after correction for the primary inner filter effect and for the primary and secondary inner filter effects is given in Panel B (curves 1 and 2, respectively). For comparison, the dependence of the total fluorescence on absorbance for NATA is given by the green curve (*q* = 0.14 [21]). Insets: Fluorescence spectra of ATTO-425 in solutions with different absorbance values (black curve 1–0.1, 2; dark red curve 2–0.2, dark green curve 3–0.5, dark yellow curve 4–0.7, dark blue curve 5–0.9, dark violet curve 6–2.8, dark blue curve 7–5.7, pink curve 8–9.5, red curve 9–13.9, green curve 10–20.1, blue curve 11–29.4): recorded (Panel A) and corrected for primary inner filter effect (Panel B).

The character of the recorded dependence of the ATTO-425 (*q* = 0.9) total fluorescence intensity on *A_FL_* (Figure 4, Panel A) differs significantly from that of NATA, which coincides with the dependence calculated according to Eq.2 (Figure 2), as was shown above. The total fluorescence intensity (and the fluorescence intensity at the maximum of the spectra; Figure 4, Panel A, Inset) increases as the absorbance increases until *A_FL_* = 2.8 and then decreases. After the correction of the total fluorescence intensity for the primary inner filter effect, the dependence of the fluorescence spectra on the solution absorbance significantly simplifies: as the absorbance increases, a red shift of the fluorescence spectra and an increase in the fluorescence intensity at the spectra maximum are observed (Figure 4, Panel B, Inset). The dependence of the corrected total fluorescence intensity on the solution absorbance increases monotonically (Figure 4, Panel B, curve 1). Nonetheless, this dependence does not coincide with that calculated according to Eq. 3 (Figure 4, curve 1). Several reasons of this effects are follows:

1. reabsorption of fluorescence light due to the overlap of the long wavelength band of the absorption spectrum and the fluorescence spectrum of the dye. This effect, called the secondary inner filter effect may underestimate the amount of the total fluorescence intensity and the greater, the larger is the absorbance of the solution;
2. interaction of dye molecules in the ground state with dimer formation;
3. interaction of dye molecules in the excited state with that in the ground state with excimer formation;
4. fluorescence quenching, caused by collision the dye molecules in the excited state with dye molecules in the ground state, acting as fluorescence quenchers;
5. clustering of the dye molecules, which leads to the fact that microenvironment of the excited dye molecule is formed not only by solvent molecules, but by a mixture of the solvent molecules and the dye molecules in the ground state, or to the fact that the quenching can be observed at low concentrations of the dye when the quenching due to collisions the molecules in their random distribution is extremely unlikely;
6. so-called effect of concentration resonance self-quenching of fluorescence, which was open by Perrin and studied Forster, Vavilov and Galanin [31]–[34].

#### Fluorescence reabsorbtion. Secondary inner filter effect

**Figure 5:**
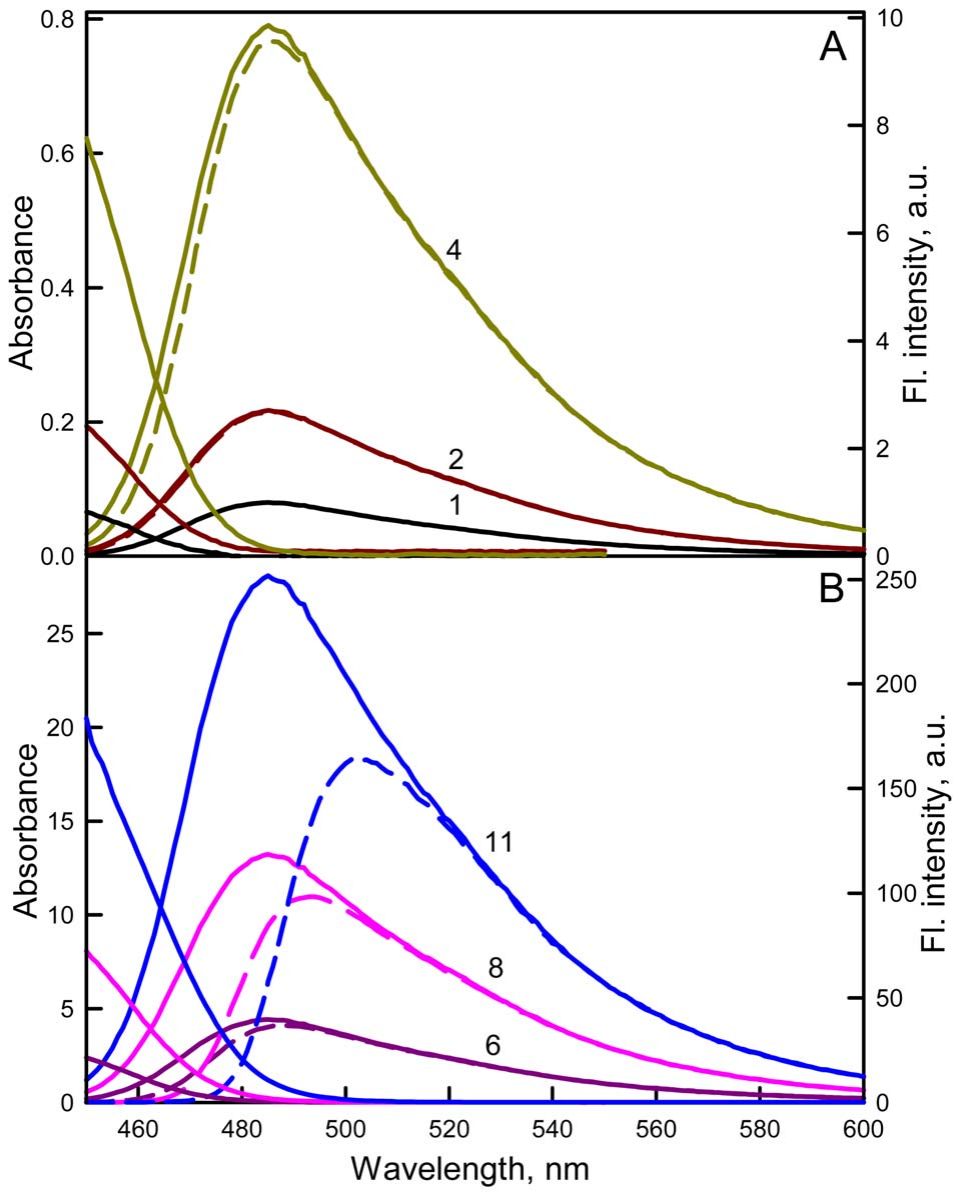
Correction of the fluorescence of the dye ATTO-425 on the secondary inner filter effect. The dashed lines show the fluorescence spectra of ATTO-425 in solutions of its different concentration corrected for primary inner filter effect. Solid lines are the fluorescence spectra measured for a solution with absorbance 0.1 (i.e., where the effect of reabsorption of the fluorescence light can be neglected) and normalized so that at a wavelength of 550 nm (where the absorption spectrum and fluorescence spectrun do not overlap) the its intensity coincided with measured fluorescence intensity of solution with given concentration, corrected for the primary inner filter effect. This figure also shows the long wavelength parts of absorption spectra (the left ordinary axis). Colors and numbers on the curves are the same as in Figure 4.

Considering the above said reasons of the deviation the dependence of total fluorescence intensity experimentally recorded and corrected for the primary inner filter effect from of the dependence calculated using Eq. 3 we, first of all, tried to take into account fluorescence reabsorption, i.e. the secondary inner filter effect because the long wavelength of ATTO-425 absorption spectra significantly overlap with the short wavelength fluorescent spectra at high dye concentration (Figure 5). We assumed that fluorescence spectra of solutions with low concentration (*A_FL_* = 0.1) are not disturbed by reabsorption, the fluorescence spectra of solutions with larger concentrations are disturbed by reabsorption at short wavelength range where fluorescence spectra overlap with absorption spectrum but not at the long wavelength range. So for fluorescence spectrum of solution with definite concentration were plotted spectrum which has the shape the same as fluorescence spectra of solution with low concentration (*A_FL_* = 0.1) with fluorescence intensity at 550 nm equal to the experimentally recorded value. It appeared that plotted in such way spectra coincide with experimentally recorded spectra not only at 550 nm but in all long wavelength range where the role of reabsorbtion is negligible. It means that for ATTO-425 reabsorption is the only reason of spectra deformation and plotted in such way fluorescence spectra can be used for plotting the dependence of total fluorescence corrected for secondary inner filter effect on absorbance (Figure 4 Panel B, curve 2). If the form of fluorescence spectra constructed in such way would deviate from the form of experimentally recorded spectra in the wave range where there is no reabsorption it means that there are some interactions of molecules in the excited state (e.g. formation of excimer or clusterization) which contributes to fluorescence spectrum deformation. In this case the correction for secondary inner filter effect will be more difficult, but it is not the case of ATTO-425. Then corrected for secondary inner filter effect fluorescence spectra were used for determination of the total fluorescence and plotting the dependence of total fluorescence on solution absorbance (Figure 4, Panel B, curve 2). It is appeared that this dependence approaches to the dependence calculated according to Eq. 3 but does not coincide with it.

#### Whether dye molecules form dimers or excimers?

**Figure 6:**
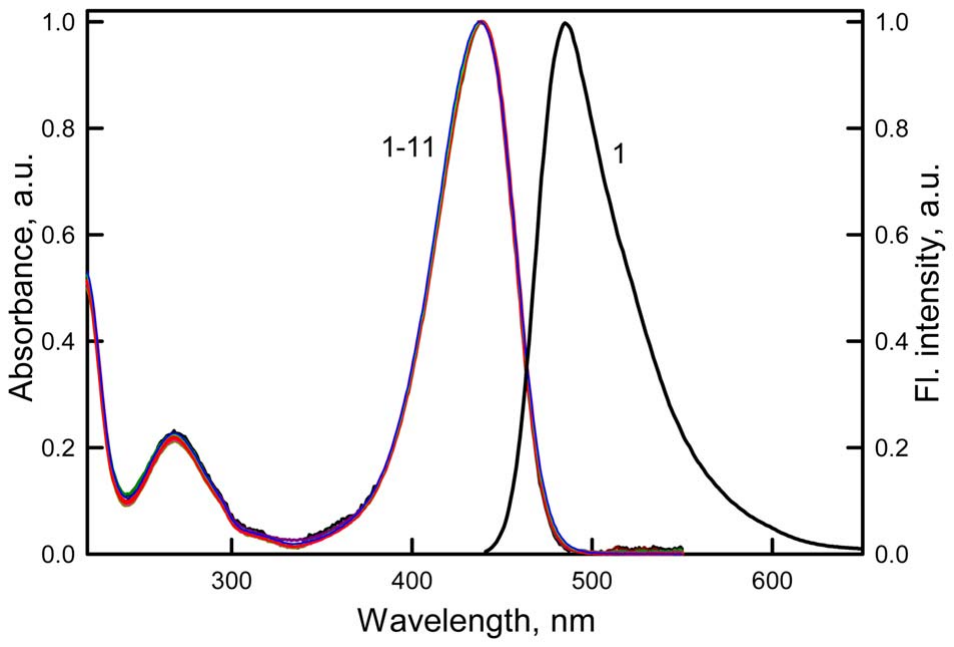
Normalized absorption and fluorescence spectrum of ATTO-425. Colors and numbers on the curves are the same as in Figure 4.

**Figure 7:**
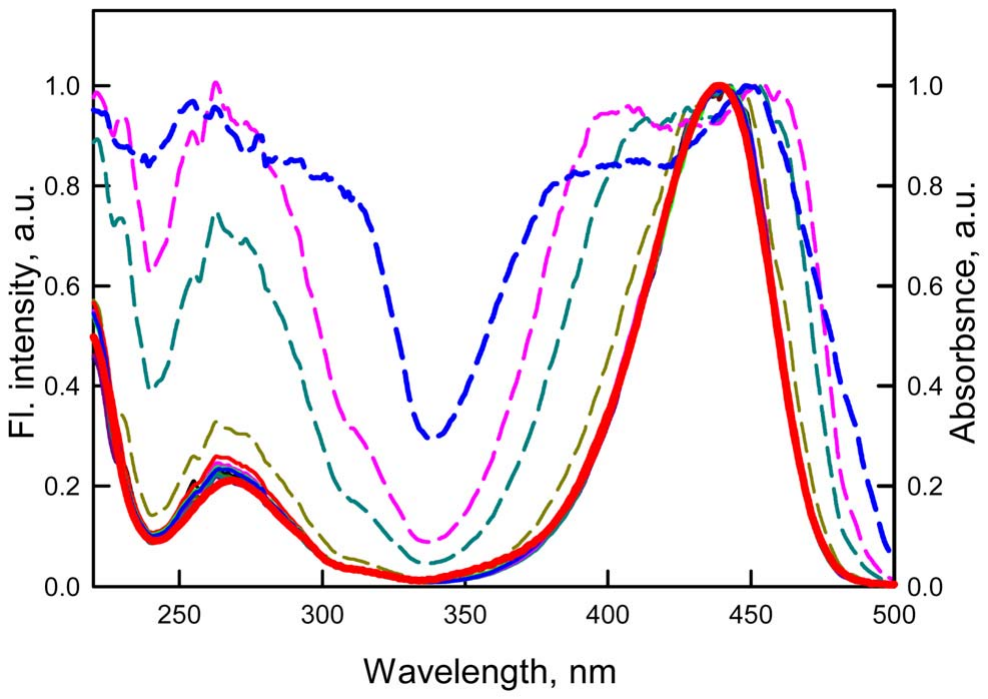
Normalized fluorescence excitation spectra of ATTO-425 solutions with different absorbance values before (dotted lines) and after (solid lines) correction for the primary inner filter effect (*λ_em_* = 510 nm). Colors and numbers on the curves are the same as in Figure 4. The normalized absorption spectrum is also given (red bold curve).

To determine why the correction for the primary and secondary inner filter effect does not linearize the dependence of the fluorescence intensity on the solution absorbance, we decided to check whether the discrepancy is connected to the interaction of ATTO-425 molecules with each other in the ground state, i.e. with dimer formation. Thus, we measured the absorption spectra and the fluorescence excitation spectra of the dye solutions at different concentrations. It was found that the form and position of the absorption spectrum of ATTO-425, as well as the molar extinction coefficient (*ε_436_* = 4.5•10^4^ M^−1^ cm^−1^), did not depend on the concentration of the dye in the range from 2.2•10^−6^ to 6.44•10^−4^ M (Figure 6). These experiments were performed using cells with different optical path lengths (but not in one cell using dilutions of the solutions). The fluorescence excitation spectra of the dye solutions were recorded at different wavelengths in the range from 470 nm to 550 nm with a step of 10 nm for the dye concentrations from 2.2 • 10^−6^ to 6.44 • 10^−4^ M and were corrected for the primary inner filter effect; these spectra coincided with each other and with the absorption spectrum (Figure 7). Figure 7 shows the experimentally recorded fluorescence excitation spectra and those after correction on primary inner filter effect. This figure shows that after correction on primary inner filter effect the fluorescence excitation spectra coincide with absorption spectra. Furthermore, as it was shown above the form of fluorescence spectra does not change with absorbance increase. Taken together, the results of measurements of the absorption and fluorescence excitation of ATTO-425 solutions indicate that at dye concentrations up to 6.44 • 10^−4^ M, the dye molecules do not form neither dimers nor excimers.

#### Self-quenching of the dye molecules fluorescence

Usually the fluorescence quenching due to collisions is observed at the quencher concentration of a fraction of mole. A calculation assuming a random distribution of molecules in solution for a dye concentration of 6.44•10^−4^ M using the Poisson equation [35] showed that the probability of finding two ATTO-425 molecules in a sphere with a radius equal to the long axis of the molecule is 10^−5^. Thus the quenching due to collision is not probable. In principal one can assume that the dye molecules are clustered if interaction with each other energetically is more favorable than with solvent molecules. The clustering can result in the fact that self-quenching can occur at lower concentrations than that for random dye distribution. Clustering of dye molecules must change the properties of their microenvironment and consequently its spectral properties. However, absorption and fluorescence spectra of the dye in the concentration range up to 6.44 • 10^−4^ M remain unchanged. Therefore, fluorescence quenching due to clustering is unlikely.

The most probable reason of the deviation of the experimentally recorded and corrected for praimary and secondary inner filter effects dependence of fluorescence intensity on solution absorbance from the dependence calculated by Eq. 3 is the effect of resonance self-quenching. In resonance quenching excitation energy transfer of the excited molecule to molecule quencher (unexcited dye molecule) can occur without collisions. Resonance self-quenching mainly dependents on the overlap of the absorption and fluorescence spectra of dyes. Some of these energy transfers is accompanied by quenching, which explains the decrease in the fluorescence quantum yield with the increase of the dye concentration in solution [31]–[34].

### Conclusion

The present work proposes a method for correcting experimentally recorded total fluorescence intensity. This method can extract the informative component of fluorescence intensity: the product of the absorbance and the fluorescence quantum yield of the object. When recorded in a spectrofluorimeter with standard geometry, the dependence of the detected fluorescence intensity on the absorbance of the fluorescent substance does not coincide with the calculated one even for small fluorophore concentrations. The use of a spectrofluorimeter with horizontal slits essentially simplifies the correction of the detected fluorescence intensity for the primary inner filter effect, allowing to use analytically determined correction coefficients. This provides a unique opportunity to work with solutions of high concentration (high absorbance) that is inherent condition for examining the photophysical properties of fluorescent dyes, the processes of fluorescence self-quenching, the formation of dimers and excimers. It is also essential for studying functionally significant interactions of biological macromolecules, in particular, for studying the interaction of proteins with their partners in the conditions of molecular crowding.
